# Sustainable Supplier Performance Evaluation and Selection with Neofuzzy TOPSIS Method

**DOI:** 10.1155/2014/434168

**Published:** 2014-10-29

**Authors:** S. K. Chaharsooghi, Mehdi Ashrafi

**Affiliations:** Industrial Engineering Department, Tarbiat Modares University, Jalal Ale Ahmad Highway, P.O. Box 14115-111, Tehran, Iran

## Abstract

Supplier selection plays an important role in the supply chain management and traditional criteria such as price, quality, and flexibility are considered for supplier performance evaluation in researches. In recent years sustainability has received more attention in the supply chain management literature with triple bottom line (TBL) describing the sustainability in supply chain management with social, environmental, and economic initiatives. This paper explores sustainability in supply chain management and examines the problem of identifying a new model for supplier selection based on extended model of TBL approach in supply chain by presenting fuzzy multicriteria method. Linguistic values of experts' subjective preferences are expressed with fuzzy numbers and Neofuzzy TOPSIS is proposed for finding the best solution of supplier selection problem. Numerical results show that the proposed model is efficient for integrating sustainability in supplier selection problem. The importance of using complimentary aspects of sustainability and Neofuzzy TOPSIS concept in sustainable supplier selection process is shown with sensitivity analysis.

## 1. Introduction

Sustainability is becoming to play an important role in supply chain management. Companies are increasingly expected to extend their sustainability efforts beyond their own operations to include those of their suppliers and to meet their customers' sustainability expectations. Traditionally, organizations consider criteria such as price, quality, flexibility, and delivery when evaluating supplier's performance. In this way companies need efficient ways to select their suppliers with regard to their sustainability policies. Now, many organizations based on the triple bottom line (TBL) approach have considered environmental, social, and economic concerns and have measured their suppliers' sustainability performance [1].

There are extended models in the literature that examine supporting facts for major dimension on TBL. Carter supposes economic, environmental, and social as major aspects and organizational culture, transparency, risk management, and strategy as supporting aspects for major dimensions in his sustainable supply chain management framework [2].

There are several evaluation models for supplier selection and evaluation in the literature. Methodologies typically found in reviews of supplier selection approaches include weighted linear model approaches, mixed integer programming, analytical hierarchy process, linear and goal programming models, matrix methods, clustering methods, human judgment models, statistical analysis, and neural networks/case-based reasoning approaches. A detailed overview of supplier selection methods can be found in [3, 4].

In this paper, given the multiple criteria nature of sustainable supplier selection problem, we propose a multicriteria method in order to evaluate sustainability performance of a suppliers based on extending TBL theory. Because human judgments and preferences are often vague and complex, and decision makers (DMs) cannot estimate their preferences with an exact scale, linguistic assessments can only be given instead of exact assessments. Therefore, fuzzy set theory is introduced into the proposed method, which is put forward to cope with such complexities.

The main contribution of this paper includes modelling the supplier selection decision problem within the context of a sustainable supply chain based on extended triple bottom line (TBL) concept.

The paper is organized as follows; the next section is a review of the related literature for sustainability in supply chain management and supplier selection by identifying the sustainability criteria that influence a company's decision in supplier selection and collaboration process. Description of fuzzy set and multiattribute decision making model used for evaluating sustainability performance of suppliers is defined in the next section. Efficiency of proposed model is shown with the numerical example in the next section and, finally, in the last section, summary and conclusion are provided.

## 2. Literature Review

In this section we focus on the sustainability supply chain management research and research dealing with supplier selection to show the different criteria used to select sustainable suppliers and the techniques being applied.

Supplier selection is a well-known phenomenon and supplier evaluation and selection problem has been studied extensively in the literature. Supplier selection process is made up by several decision making steps. Supplier selection metrics varied significantly in previous researches. Cost, quality, on time delivery, and flexibility are major factors that have been used in supplier selection literatures. Early researches showed special emphasis mainly on cost and then on reliability, responsiveness, safety, and environmental factors [5].

More recently with introducing the sustainable supply chain management (SSCM), studies have utilized more attributes beyond those used in operational decisions. SSCM is defined as the management of material and information flows as well as cooperation among organizations along the supply chain while integrating the “triple-bottom-line” factors into account. These factors include all three dimensions of sustainable development (economic, environmental, and social) [6].

The TBL approach suggests that besides economic performance, organizations need to engage in activities that positively affect the environment and the society. By adopting the triple bottom line approach, an organization takes a responsible position on economic prosperity, environmental quality, and social justice [7].

There are some supportive factors for these TBL dimension. Carter and Rogers regard organizational culture, transparency, risk management, and strategy as supporting facts for major dimensions in their sustainable supply chain management framework [8].

The result of our literature review show that Supplier selection problem is a very old problem in the operation research context and there is emphasis on environmental and social aspects besides economic aspect in supplier selection process, in recent researches. Bai and Sarkis are the pioneers in introducing the sustainability concept into the supplier selection problem. They develop a sustainability framework and utilize grey system and rough set theory in their supplier selection process [7]. Amindoust et al. determined sustainable supplier selection criteria and subcriteria and based on those criteria and subcriteria a fuzzy logic methodology is proposed onto evaluation and ranking of a given set of suppliers [9]. Govindan et al. used fuzzy TOPSIS model for their sustainable supplier selection process [10].

### 2.1. TBL Sustainable Supplier Selection Criteria

Supply chain management initiatives are reviewed in this section to determine the supplier selection criteria.

The analysis of supplier evaluation and selection criteria has been the focus of many researchers and purchasing practitioners since the 1960s. Quality, Delivery, and Performance history are the three most important criteria in supplier evaluation process [3]. There are several comprehensive reviews in supplier selection and evaluation criteria and methods that concluded price were the highest-ranked criteria, followed by delivery and quality [11]. The recent literature review of the MCDM approaches for supplier evaluation and selection showed that the most popular criteria are quality, delivery, cost, manufacturing capability, and service [12]. In another comprehensive review of criteria used for supplier selection it was shown that quality, price, and delivery performance are the most important economic supplier selection criteria [13].

Supplier selection in green supply chain management (GSCM) is mostly focused on environmental aspect of sustainability. GSCM is defined by minimizing and preferably eliminating the negative effects of the supply chain on the environment and a firm's environmental sustainability and ecological performance can be demonstrated by its suppliers. Accordingly, developing the environmental criteria is very important in GSCM [14]. Carbon management is one of the most important issues in GSCM that is considered in the literature. Hsu et al. reviewed the carbon management literature and thirteen criteria of carbon management with three dimensions were identified in his research; the obtained results show that the criteria of management systems of carbon information and training related to carbon management are revealed to be the top two significant influences in selecting suppliers with carbon management competencies [15]. Humphreys et al. introduced environmental cost, management and environmental competencies, environmental management systems design for environment, and green image as integrated criteria to green supplier selection [16]. Tseng and Chiu, in their research showed that among the supplier selection models being used, environmentally preferable bidding and life cycle assessment which assesses green purchasing impacts and their financial consequences through the entire product life cycle are the most popular criteria [17].

Importance of social aspect of sustainability in selection of international suppliers from the world's emerging economies is evident in the relevant literature. Based on stakeholder theory the pressures from the customers, the government, and the employees in the selection of emerging economy suppliers were examined and relation of such socially sustainable supplier selection to the capabilities of the firm's suppliers, its market reputation, and learning in its supply management organization is showed in [18]. The social criteria are considered fewer than other sustainability aspects in the literature. Social measures can be categorized into internal and external social criteria based on company aspect. The measures such as employment practice and safety can be classified as subcategories of internal criteria and masseurs such as local influences as subcategories of external social criteria [7]. Amindoust et al. determined five subcriteria for social dimension in the proposed supplier evaluation method that has been proposed by the literature [9]. Standards and international guidelines can be used for developing social criteria in supplier selection. One of the most important guidelines is UN global compact (UNGC), the world's largest corporate responsibility initiative with over 8000 business and nonbusiness participants in more than 140 countries. The UNGC distinguishes between four different (noneconomic) dimensions of sustainability: human rights, labour, environment, and anticorruption [19].

### 2.2. Complementary Sustainable Supplier Selection Criteria

There are some aspects of sustainability which were not included in explicit definitions. Risk management, transparency, strategy, and culture are proposed as supporting facts in TBL for sustainability [8]. The concept of risk and its management was identified as a reoccurring theme in the sustainability theory. Carter and Ragers advocates that within the context of sustainability, an organization must manage not only short-term financial results, but also risk factors such as harm resulting from its products, environmental waste, and worker and public safety. Such supply chain risks can result from natural disasters such as hurricanes, legal liabilities, poor demand forecasting, failure to coordinate demand requirements across the supply chain, fluctuating prices for key raw materials including energy, poor supplier quality, shipment quantity inaccuracies, and poor environmental and social performance by a firm and its suppliers which can result in costly legal actions. Therefore the definition of risk and risk management can be different. Within the context of sustainability, supply chain risk management is defined as the ability of a firm to understand and manage its economic, environmental, and social risks in the supply chain [8].

Transparency is another supporting fact for TBL that has been mentioned extensively within discussions of organizational sustainability. It is being driven, in part, by the rapid speed of communication via the internet and globalization of supply chains which have led to a “flat world.” Transparency includes not only reporting to stakeholders, but also actively engaging stakeholders and using their feedback and input to both secure buy-in and improve supply chain processes. This transparency encompasses green marketing activities within a stakeholder perspective [8].

The last supporting facts of TBL are strategy and culture. An organization's sustainability initiatives and its corporate strategy must be closely interwoven, rather than separate programs that are managed independently of one another. Organizations that become sustainable enterprises do not simply overlay sustainability initiatives with corporate strategies. These organizations also have (or have changed) their company cultures and mindsets [8].

There are various approaches to address the supplier selection criteria and interpretation of them in a variety of ways. We selected some representative criteria from extending TBL framework and combined subcriteria applied by these researchers into main sustainable criteria although it is clear that these criteria are not meant to thoroughly describe the sustainable performance of a supplier in general but rather to serve as an example of the measures that could establish a number of criteria and those that could be considered in the literature from a sustainability perspective. The sustainability supplier selection criteria are summarized in Table 1.

### 2.3. Fuzzy Sets Theory

In 1965, fuzzy sets were proposed to confront the problems of linguistic or uncertain information and to be a generalization of conventional set theory. The fusion of MCDM and fuzzy set theory strengthen a new decision theory which was later being known as Fuzzy MCDM [20].

In fuzzy sets, a fuzzy number is a generalization of a regular, real number in the sense that it does not refer to one single value but rather to a connected set of possible values, where each possible value has its own weight between 0 and 1 and this weight is called the membership function. In this paper triangular fuzzy numbers are used to assess the preferences of DMs. The reason for using a triangular fuzzy number is that it is intuitively easy for the DMs to use and calculation. A triangular fuzzy number can be shown as (*l*, *m*, *u*) where *l* and *u* stand for the lower and upper bounds of the fuzzy number, respectively, and *m* for the modal value.

**Definition 1 .**

**Figure 1:**
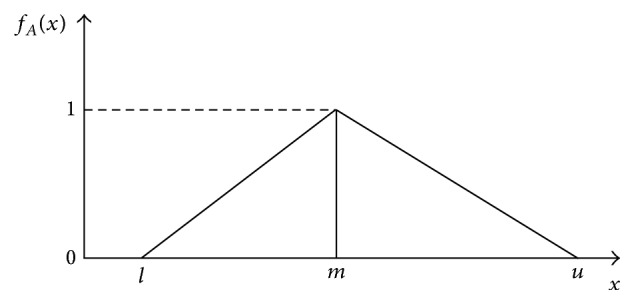
Memberships function of triangular fuzzy number *A*.

The membership function of the fuzzy number *f*_*A*_(*x*) is defined (see Figure 1) as

(1) $$\begin{array}{c} f_{A}\left(x\right)=\{\begin{array}{cc} \frac{x-l}{m-l}, & l\leq x\leq m\\ \frac{u-x}{u-m}, & m\leq x\leq u\\ 0, & \text{otherwise}. \end{array} \end{array}$$

**Definition 2 .**

Let *A* = (*l*_1_, *m*_1_, *u*_1_) and *B* = (*l*_2_, *m*_2_, *u*_2_) be two triangular fuzzy numbers. Then the operational laws of these two triangular fuzzy numbers are as follows:

(2) A(+)B=(l1,m1,u1)(+)(l2,m2,u2)=(l1+l2,m1+m2,u1+u2),A(−)B=(l1,m1,u1)(−)(l2,m2,u2)=(l1−l2,m1−m2,u1−u2),A(×)B=(l1,m1,u1)(×)(l2,m2,u2)=(l1×l2,m1×m2,u1×u2),A(/)B=(l1,m1,u1)(/)(l2,m2,u2)=(l1l2,m1m2,u1u2),K×A=(K×l1,K×m1,K×u1),1(A)=(1u1,1m1,1l1),d(A,B)=13[(l1−l2)2+(m1−m2)2+(u1−u2)2],

where *d*(*A*, *B*) is the distance between fuzzy numbers *A*,  *B*.

**Definition 3 .**

In a decision group that has *K* DMs, with a positive triangular fuzzy number *R*_*k*_, *k* = 1,…, *k* and *f*_*Rk*_(*x*) as fuzzy rating of each DM and membership function, respectively, the aggregated fuzzy rating can be defined as

(3) $$\begin{array}{c} R=\left(l,m,u\right),\;\;k=1,2,\ldots ,k, \end{array}$$

where *l* = min⁡_*k*_⁡*l*_*k*_, *m* = (1/*k*)∑*m*_*k*_, *u* = max⁡_*k*_⁡*u*_*k*_.

## 3. The Proposed Neofuzzy TOPSIS Method

Multicriteria group decision making problems are frequently encountered in practice. Several methods exist that can be applied to solve such problems and among these methods the idea of technique for order preferences by similarity to an ideal solution (TOPSIS) method is very straightforward. The classical TOPSIS proposed by Hwang and Yoon is based on the idea that the best alternative should have the shortest distance from the positive ideal solution and the greatest distance from the negative one.

As mentioned in [10] TOPSIS advantages make it a major MADM technique as compared to other related techniques such as analytical hierarchical process (AHP) and ELECTRE.

TOPSIS is a powerful technique but it has a big weakness that is the fact that it does not provide us with a good alternative. According to this technique, the nearest alternative to the ideal solution is a suitable one and the ideal solution origins from the information of the available alternatives. In the sustainability application there is no assurance that the available alternatives are unsuitable condition for minimum qualification especially in environment and social issues. To achieve sustainable supply chain, it is necessary to define sustainability standards, frameworks, and minimum requirements for suppliers and to improve these reference levels continually.

In the Neo-TOPSIS two absolute (bad and good) candidates are inserted in the decision maker (DM) matrix. These two absolute candidates are maximum and minimum standards of a decision maker. Neo-TOPSIS compares candidates (suppliers) with these two standards, so the distance between the candidates becomes real [21].

The TOPSIS solution method can be defined by the following steps.

**Step 1 .**

Calculate the normalized decision matrix. The normalized fuzzy-decision matrix can be represented as

(4) $$\begin{array}{c} R={\left[r_{ij}\right]}_{m\times n}, \end{array}$$

where

(5) rij=(lijUj∗,mijUj∗,uijUj∗), j∈B,Uj∗=max⁡i⁡ uij, j∈B,rij=(lj−lij,lj−mij,lj−uij), j∈C,lj−=min⁡i⁡ lij, j∈C.

In (5), *B* and *C* represent the sets of benefit and cost criteria, respectively.

**Step 2 .**

Calculate the weighted normalized decision matrix. The weighted normalized fuzzy decision matrix *V* is computed by multiplying the weights *w*_*j*_ of evaluation criteria by the normalized fuzzy decision matrix *r*_*ij*_

(6) $$\begin{array}{c} V={\left[v_{ij}\right]}_{m*n}, \end{array}$$

where *v*_*ij*_ = *r*_*ij*_ · *w*_*j*_ and *w*_*j*_ is the weight of the *j*th attribute or criterion.

**Step 3 .**

Determine the Neo positive- and negative-absolute candidates: the Neofuzzy positive-absolute candidate (FPAC, *A* ^+^) and Neofuzzy negative-absolute candidate (FNAC, *A* ^−^) can be defined as

(7) $$\begin{array}{c} A^{+}=\left(v_{1}^{+},v_{2}^{+},\ldots ,v_{n}^{+}\right),\\ A^{-}=\left(v_{1}^{-},v_{2}^{-},\ldots ,v_{n}^{-}\right), \end{array}$$

where *v*_*j*_^+^ = (max⁡_*i*_⁡{*v*_*ij*3_})(1 + *N*_max⁡_) and *v*_*j*_^−^ = (min⁡_*i*_⁡{*v*_*ij*1_})(1 − *M*_min⁡_)  *i* = 1,…, *m*,  *j* = 1,…, *n*.

In (7) *N*_max⁡_ and *M*_min⁡_ are the quantity of increasing and decreasing in number of *r*_*ij*_.

**Step 4 .**

Determine the distance of each alternative from the positive and negative absolute candidates that can be calculated as

(8) $$\begin{array}{c} d_{i}^{+}=\underset{j}{\sum }d\left(v_{ij},v_{j}^{+}\right)\;i=1,\ldots ,m,\\ d_{i}^{-}=\underset{j}{\sum }d\left(v_{ij},v_{j}^{-}\right)\;i=1,\ldots ,m. \end{array}$$

**Step 5 .**

Calculate the relative closeness to the ideal solution. A closeness coefficient is defined to determine the ranking order of all possible suppliers after *d*_*i*_^+^ and *d*_*i*_^−^ of each alternative *A*_*i*_  (*i* = 1,2,…, *m*) have been calculated. The closeness coefficient (CC) of each alternative is calculated as

(9) $$\begin{array}{c} {\text{RC}}_{i}=\frac{d_{i}^{-}}{\left(d_{i}^{+}+d_{i}^{-}\right)},\;i=1,\ldots ,m. \end{array}$$

**Step 6 .**

Rank the preference order. Alternative *A*_*i*_ is closer to the FPAC (*A* ^+^) and farther from FNAC (*A* ^−^) as RC approaches to 1.

According to the descending order of RC we can determine the ranking order of all alternatives and select the best possible one.

## 4. Numerical Example

**Figure 2:**
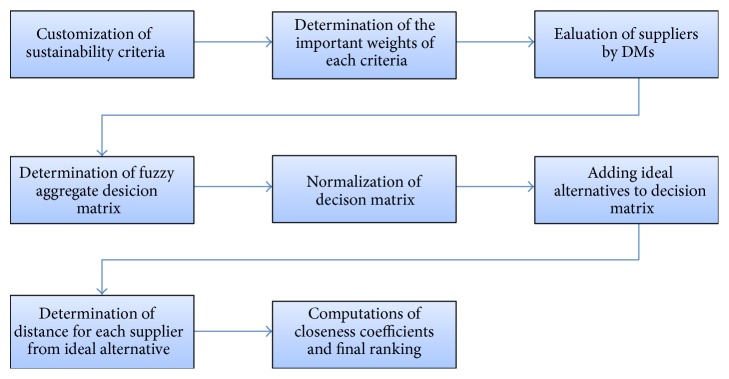
The sustainability supplier selection procedure.

To examine the practicality and the effectiveness of the proposed approach for supplier selection and evaluation, numerical example is illustrated for evaluating sustainability performance of suppliers in the oil and petroleum industry case in Iran. The sustainability supplier selection procedure is illustrated in Figure 2. At first we develop a sustainability evaluation framework with criteria illustrated in Table 1. We will assume two subfactors for each of the main sustainability pillars. Therefore, we have two environmental attributes, Ev1, Ev2; two economic/business attributes Ec1, Ec2; two social attributes So1, So2; two transparencies attribute Tr1, Tr2; two risk management Rm1, Rm2; two organizational an cultural attributes Cu1, Cu2. The Ec criteria are cost and benefit.

An operations manager (DM1), a financial manager (DM2), a purchasing manager (DM3), and an environmental manager (DM4) will be considered as four decision makers in the decision making process. The relative importance weights and the ratings important of the criteria which have been described using linguistic variables are defined in Table 2. The results of importance weights of the criteria and the ratings of each supplier with respect to the twelve criteria are shown in Tables 3, 4, and 5.

Normalized fuzzy decision matrix is computed with (5), and then fuzzy weighted decision matrix is constructed using (6) and the result is illustrated in Table 6.

The distance of each supplier from FPAC and FNAC with respect to each criterion and the closeness coefficient of each supplier are computed with (8) and the results are provided, respectively, in Table 7.

Using the distances *d*(*A*_*i*_, *A* ^+^) and *d*(*A*_*i*_, *A* ^−^), we compute the closeness coefficient for the alternatives using (9) and the final results are shown in Table 8.

### 4.1. Sensitivity Analysis

To investigate the impact of decision criteria in the final suppliers ranking we constructed a sensitivity analysis. This inquiry is useful in situations where uncertainties exist in the definition of the importance of different factors and situations. In the first steps, the importance of adding complementary sustainability criteria to the selection model was attended to in the final ranking solution. In the second step the importance of determination of *N*_min⁡_, *M*_max⁡_ in FNAC and FPAC calculation is considered. For this purpose, three different scenarios are considered. In economy focused scenario, ideal alternative is determined with rigorous emphasis on economy dimension and economic criteria involved in CC_*i*_ calculations while, in environment and social scenarios; the environmental and social dimensions of ideal alternative is considered and the relevant criteria are involved in CC_*i*_ calculations.

**Figure 3:**
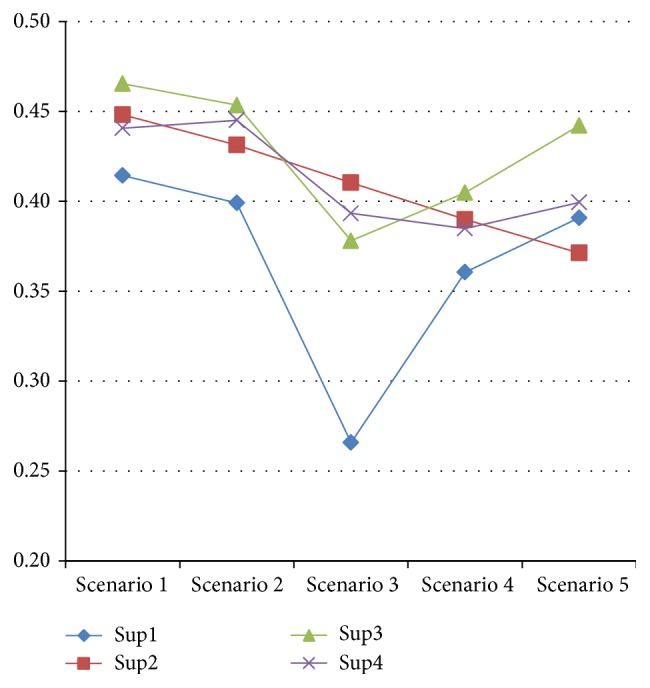
Sensitivity analysis result.

The details of five scenarios are presented in Table 9, and Figure 3 illustrates a graphical representation of these results. It can be seen that for two primary scenarios supplier 3 has emerged as the best supplier. It can be perceived that the sustainable supplier selection decision is relatively insensitive to complementary criteria; however when the complementary criteria get involved in the problem the ranking of suppliers 2 and 3 is changed.

Last three scenarios show the applicability of Neofuzzy TOPSISS method in the sustainable supplier selections. Proposed method showed that with changing the definition of ideal alternative with respect to sustainability dimensions, different changes in suppliers ranking would be observed. Finally it seems that supplier 3 has good performance assessment in different situations and it is best to choose it as the best supplier. On the other hand, supplier 2 has a stable behavior among the different scenarios and it seems that supplier 2 is a more wise selection in the business environments where uncertainties exist.

## 5. Conclusion

This paper focused mainly on the integrating complementary criteria to TBL sustainability factors for supplier evaluation. A comprehensive analysis of sustainable business operations should consider all dimensions simultaneously.

In this paper we introduced a fuzzy MCDM approach for supplier selection decisions with consideration of sustainability criteria and a numerical example was presented to exemplify the proposed method. First, the criteria for evaluating sustainable performance were identified based on the literature. Second, the linguistic ratings to the criteria and the alternatives were determined, and Neofuzzy TOPSIS was used to aggregate the ratings and to generate an overall performance score by which we measured the sustainable performance of each supplier. Determining the ideal alternative in Neofuzzy TOPSISS based on the best practices and standards instead of performance evaluations of existing suppliers improved the efficiency and applicability of the proposed method in sustainability context. Finally, we performed sensitivity analysis to determine the influence of different changes and situations on the decision making process.

The proposed method has many advantages for sustainability and supply chain management practitioners. First, with linguistics variables and fuzzy MADM method introduced in this paper, the proposed approach can be used in real word sustainability problems with more efficiency. Second, companies can use the proposed method for periodic supplier's assessments and also for designing their improvement plans. Third, the Neo TOPSIS concept used in the proposed method increases the applicability of the methods in sustainability applications. In the first steps of sustainability journey, many aspects of sustainability, especially social and environmental criteria may be missed by suppliers. Therefore, Neo TOPSIS concept and involving best practice and standard frameworks of the ideal alternative to existing ideal performance alternative, avoid the bias of decision makers' choices to a specific dimension of sustainability and finally, based on implementation of these sustainable supplier evaluation, companies can identify and prioritize opportunities for improving their sustainability performances in a holistic view rather than the traditional TBL approach, which may lead to a reduction in the negative environmental and social impact of their activities.

One of the limitations of the paper is that we have introduced a hypothetical illustrative example rather than providing a real world application. Practical questions pertaining to the validity and accuracy of these decisions would need to be investigated for operational feasibility of this methodology. The availability of the information and data needed for the application of the methodology is one of the limitations to its operational feasibility. This study may be the subject of future research. Dynamic evaluation models that are able to integrate the selection phase with monitoring and continuous analysis of the supplier selection can be investigated. In addition, order quantity allocation, after ranking all suppliers, is another important issue that could become a new trend in the future.

## Figures and Tables

**Table 1:** Sustainability supplier selection criteriaSustainability dimension.

	Criteria	Definition
Economy	Cost [3, 12, 22, 23]	Cost of acquisitioning product, including product, inventory, logistic and …
	Technology capability [24, 25]	Technology and capability of the supplier to meet current and future demand of the firm
	Quality [26–28]	Meet the quality requirements
	Delivery [3, 4]	Ability of to fulfill shipping orders within the period of time promised
	Service apability [21, 29, 30]	Ability to provide added service value
	Flexibility [31–33]	Ability to tolerate the variability
	Financial capability [34, 35]	Economic stability and long-term financial health of supplier

Environment	Pollution production [7, 9, 36]	Air emission pollutant, waste water, solid wastes and harmful materials release
	Resource consumption [36, 37]	Resource consumption in terms of raw material, energy, and water
	Environmental management system [37, 38]	Establishment of environmental commitment and policy, certifications, planning and control of environmental activities
	Eco-design [9]	Design of products for reduced consumption of material/energy, design of products for reuse, recycle, recovery of material, design of products to avoid or reduce use of hazardous material

Social	Employment practices [7, 10]	The interests and rights of employees
	Health and safety [7, 9]	Work safety and labour health
	Local communities influence [39]	Relationship with stakeholders like local communities and non-governmental organizations (NGOs)
	Contractual stakeholders influence [18]	Relationship with contractual stakeholders like suppliers and customers

Risk management system	Risk analysis [40, 41]	Examination of sustainability risk in various degrees of detail
	Risk evaluation [42]	Consideration of consequence of issues and prioritization of them
	Risk management [40]	Decision making process to how best to deal with risks

Transparency	Communication [8]	Communication openness
	Financial [8]	Timely, meaningful and reliable disclosures about a company's financial performance

Culture and strategy	Relationship [43]	Strategy of supplier in relationships such as long term relationships
	Management capability [44]	Capability of top management systems of supplier and strategic fit
	Organizational structure [8]	Agility in organizational structure and personnel

**Table 2:** Linguistic variable for the rating and relative importance weights of criteria [17].

Linguistic variable for relative importance weights of criteria		Linguistic variable for rating	
Linguistic variable	Fuzzy numbers	Linguistic variable	Fuzzy numbers
Very low (VL)	(0.1, 0.1, 0.3)	Very Poor (VP)	(1, 1, 3)
Low (L)	(0.1, 0.3, 0.5)	Poor (P)	(1, 3, 5)
Medium (M)	(0.3, 0.5, 0.7)	Fair (F)	(3, 5, 7)
High (H)	(0.5, 0.7, 0.9)	Good (G)	(5, 7, 9)
Very high (VH)	(0.7, 0.9, 0.9)	Very Good (VG)	(7, 9, 9)

**Table 3:** Importance weights of the criteria from three DMs.

DMs	Economy criteria		Environment criteria		Social criteria		Risk management criteria		Transparency criteria		Culture criteria	
	Ec1	Ec2	En1	En2	So1	So2	Rm1	Rm2	Tr1	Tr2	Cu1	C2
Dm1	H	M	M	VH	M	L	VH	H	L	M	M	H
Dm2	VH	H	M	VH	M	VL	VH	H	VL	M	H	VH
Dm3	VH	VH	H	H	H	L	VH	VH	M	M	H	VH
Dm4	H	M	M	M	VH	VL	H	H	L	L	M	H

**Table 4:** Evaluation of suppliers on sustainability criteria by DMS.

	Economy criteria		Environment criteria		Social criteria		Risk management criteria		Transparency criteria		Culture criteria	
	Ec1	Ec2	En1	En2	So1	So2	Rm1	Rm2	Tr1	Tr2	Cu1	C2
DM1												
Sup1	F	VG	VG	F	VP	G	F	VP	P	VG	VP	P
Sup2	F	F	VP	VP	G	G	P	VG	F	G	P	F
Sup3	G	P	P	P	VG	F	P	VP	G	P	VP	F
Sup4	F	G	G	VG	F	F	VG	P	P	G	F	F
DM2												
Sup1	P	VP	VG	VP	F	G	F	P	G	P	P	F
Sup2	VP	P	VP	G	G	VP	VG	P	F	F	P	VG
Sup3	F	VP	P	VP	G	VG	F	G	VG	G	P	VP
Sup4	P	F	F	P	VP	VG	P	G	G	G	F	P
DM3												
Sup1	VP	G	VG	G	VG	VG	F	G	F	F	VP	VP
Sup2	G	VG	P	VG	P	VP	G	P	G	F	VG	P
Sup3	G	VP	G	F	F	VG	VP	VP	F	VG	P	VG
Sup4	F	F	VG	G	G	F	VG	G	P	VG	F	G
DM4												
Sup1	G	P	P	G	F	F	VP	G	P	VP	F	G
Sup2	VP	P	VG	VP	VP	VG	F	VP	P	F	P	F
Sup3	G	F	VP	G	F	VG	F	P	VP	G	G	P
Sup4	P	VP	VP	VG	G	VG	VP	G	VG	VG	VP	P

**Table 5:** Fuzzy aggregated decision matrix and fuzzy weights of criteria.

	Ec1			Ec2			En1			En2			So1			So2		
Weight	0.7	0.7	0.9	0.7	0.7	0.9	0.1	0.1	0.3	0.5	0.7	0.9	0.3	0.5	0.7	0.3	0.5	0.7
Sup1	3	5.5	9	1	6	9	1	5	9	1	4.5	9	1	5.5	9	3	6	9
Sup2	1	3	7	1	2.5	7	1	4.5	9	1	3	9	1	5	9	1	6.5	9
Sup3	1	5	9	1	5.5	9	1	7	9	3	7	9	1	6	9	1	6	9
Sup4	1	4.5	9	1	3	7	1	3.5	9	1	6	9	1	4.5	9	3	8	9

	Rm1			Rm2			Tr1			Tr2			Cu1			C2		
Weight	0.5	0.7	0.9	0.5	0.7	0.9	0.1	0.1	0.3	0.1	0.3	0.5	0.5	0.7	0.9	0.5	0.7	0.9

Sup1	1	5	9	1	3.5	9	1	4.5	9	1	6.5	9	1	2.5	7	1	4.5	7
Sup2	1	5.5	9	1	5	9	3	7	9	1	5.5	9	1	3.5	7	1	4.5	9
Sup3	1	5.5	9	1	4.5	9	1	5	9	3	7	9	1	4.5	9	1	5	9
Sup4	1	3	7	1	4.5	9	1	4	9	1	5.5	9	1	4	9	1	4.5	9

**Table 6:** Weighted normalized fuzzy decision matrix.

	Ec1			Ec2			En1			En2			So1			So2		
Sup1	0.078	0.1	0.23	0.08	0.12	0.7	0.1	0.4	0.7	0.1	0.4	0.7	0.1	0.4	0.7	0.2	0.5	0.7
Sup2	0.1	0.2	0.7	0.1	0.28	0.7	0.1	0.4	0.7	0.1	0.2	0.7	0.1	0.4	0.7	0.1	0.5	0.7
Sup3	0.078	0.1	0.7	0.08	0.13	0.7	0.1	0.5	0.7	0.2	0.5	0.7	0.1	0.5	0.7	0.1	0.5	0.7
Sup4	0.078	0.2	0.7	0.1	0.23	0.7	0.1	0.3	0.7	0.1	0.5	0.7	0.1	0.4	0.7	0.2	0.6	0.7

	Rm1			Rm2			Tr1			Tr2			Cu1			C2		

Sup1	0.1	0.4	0.7	0.08	0.3	0.7	0.1	0.4	0.7	0.08	0.5	0.7	0.08	0.2	0.54	0.08	0.4	0.54
Sup2	0.1	0.4	0.7	0.08	0.4	0.7	0.2	0.5	0.7	0.08	0.4	0.7	0.08	0.3	0.54	0.08	0.4	0.7
Sup3	0.1	0.4	0.7	0.08	0.4	0.7	0.1	0.4	0.7	0.23	0.5	0.7	0.08	0.4	0.7	0.08	0.4	0.7
Sup4	0.1	0.2	0.5	0.08	0.4	0.7	0.1	0.3	0.7	0.08	0.4	0.7	0.08	0.3	0.7	0.08	0.4	0.7

**Table 7:** Distances between suppliers and *A* ^+^, *A* ^−^ with respect to each criterion.

	Ec1	Ec2	En1	En2	So1	So2	Rm1	Rm2	Tr1	Tr2	Cu1	Cu2
*N*_min⁡_/*M*_max⁡_	0.1	0.2	0.05	0.5	0.1	0.2	0.15	0.2	0.1	0.05	0.15	0.1
*d*(sup1, *A* ^+^)	0.63	0.61	0.43	0.72	0.45	0.42	0.49	0.55	0.47	0.40	0.57	0.49
*d*(sup2, *A* ^+^)	0.50	0.54	0.44	0.76	0.46	0.49	0.48	0.52	0.34	0.42	0.54	0.47
*d*(sup3, *A* ^+^)	0.54	0.61	0.40	0.59	0.44	0.50	0.48	0.53	0.46	0.31	0.50	0.46
*d*(sup4, *A* ^+^)	0.54	0.56	0.46	0.69	0.47	0.38	0.55	0.53	0.48	0.42	0.51	0.47
*d*(sup1, *A* ^−^)	0.10	0.37	0.40	0.42	0.42	0.45	0.41	0.39	0.40	0.44	0.29	0.32
*d*(sup2, *A* ^−^)	0.38	0.39	0.40	0.40	0.41	0.45	0.42	0.41	0.47	0.42	0.30	0.40
*d*(sup3, *A* ^−^)	0.37	0.37	0.45	0.49	0.43	0.44	0.42	0.40	0.41	0.46	0.40	0.41
*d*(sup4, *A* ^−^)	0.37	0.38	0.38	0.46	0.40	0.50	0.29	0.40	0.39	0.42	0.39	0.40

**Table 8:** Computations of closeness coefficients and final ranking of suppliers.

	*d* ^+^	*d* ^−^	CC_*i*_	Rank
Sup1	6.22	4.40	0.41	4
Sup2	5.95	4.83	0.45	2
Sup3	5.80	5.05	0.47	1
Sup4	6.06	4.77	0.44	3

**Table 9:** Results of sensitivity analysis of Neofuzzy TOPSIS method for sustainable supplier selection.

Scenarios	Criteria	*N*_min⁡_, *M*_max⁡_	Suppliers ranking
Scenario1	TBL and complementary criteria	Balanced	3 > 2 > 4 > 1
Scenario2	TBL Criteria	Balanced	3 > 4 > 2 > 1
Scenario3	TBL and complementary criteria	Economy focused	2 > 4 > 3 > 1
Scenario4	TBL and complementary criteria	Environmental focused	3 > 2 > 4 > 1
Scenario5	TBL and complementary criteria	social focused	3 < 4 < 1 < 2
